# Self-Supplying Guide RNA-Mediated CRISPR/Cas12a Fluorescence System for Sensitive Detection of T4 PNKP

**DOI:** 10.3390/molecules27249019

**Published:** 2022-12-17

**Authors:** Xiuhua Yuan, Hui Yuan, Bingxin Liu, Yeling Liu

**Affiliations:** 1 School of Mechanical and Automotive Engineering, Liaocheng University, Liaocheng 252059, China; 2 Department of Chemistry, Liaocheng University, Liaocheng 252059, China

**Keywords:** T4 PNKP, CRISPR/Cas12a, transcription

## Abstract

Sensitive detection methods for T4 polynucleotide kinase/phosphatase (T4 PNKPP) are urgently required to obtain information on malignancy and thereby to provide better guidance in PNKP-related diagnostics and drug screening. Although the CRISPR/Cas12a system shows great promise in DNA-based signal amplification protocols, its guide RNAs with small molecular weight often suffer nuclease degradation during storage and utilization, resulting in reduced activation efficiency. Herein, we proposed a self-supplying guide RNA-mediated CRISPR/Cas12a system for the sensitive detection of T4 PNKP in cancer cells, in which multiple copies of guide RNA were generated by in situ transcription. In this assay, T4 PNKP was chosen as a model, and a dsDNA probe with T7 promoter region and the transcription region of guide RNA were involved. Under the action of T4 PNKP, the 5′-hydroxyl group of the dsDNA probe was converted to a phosphate group, which can be recognized and digested by Lambda Exo, resulting in dsDNA hydrolysis. The transcription template was destroyed, which resulted in the failure to generate guide RNA by the transcription pathway. Therefore, the CRISPR/Cas12a system could not be activated to effectively cleavage the F-Q-reporter, and the fluorescence signal was turned off. In the absence of T4 PNKP, the 5′-hydroxyl group of the substrate DNA cannot be digested by Lambda Exo. The intact dsDNA acts as the transcription template to generate a large amount of guide RNA. Finally, the formed Cas12a/gRNA complex triggered the reverse cleavage of Cas12a on the F-Q-reporter, resulting in a “turn-on” fluorescence signal. This strategy displayed sharp sensitivity of T4 PNKP with the limit of detection (LOD) down to 0.0017 mU/mL, which was mainly due to the multiple regulation effect of transcription amplification. In our system, the dsDNA simultaneously serves as the T4 PNKP substrate, transcription template, and Lambda Exo substrate, avoiding the need for multiple probe designs and saving costs. By integrating the target recognition, Lambda Exo activity, and trans-cleavage activity of Cas12a, CRISPR/Cas12a catalyzed the cleavage of fluorescent-labeled short-stranded DNA probes and enabled synergetic signal amplification for sensitive T4 PNKP detection. Furthermore, the T4 PNKP in cancer cells has been evaluated as a powerful tool for biomedical research and clinical diagnosis, proving a good practical application capacity.

## 1. Introduction

T4 polynucleotide kinase/phosphatase (T4 PNKP), a pivotal DNA repair enzyme that possesses both 3′-phosphatase and 5′-kinase activities, serves a critical role in maintaining the genomic stability of normal tissue cells and conferring cancer cells’ adaptive resistance to genotoxic agents [1,2]. Recent studies have confirmed that abnormalities in PNKP can lead to abnormal cell proliferation, which is closely related to the occurrence of vital human diseases, such as Rothmund–Thomson syndrome and Bloom syndrome [3,4]. Therefore, sensitive detection methods for PNKP are urgently required to obtain information on malignancy and, thereby to provide better guidance in PNKP-related disease diagnostics and drug screening.

Currently, widely available detection approaches for T4 PNKP mainly include colorimetric [5], electrochemical [6,7,8], flow cytometric [9], photoelectrochemical [10,11], and fluorescence assays [12]. Among them, fluorescence assays have gained intensive attention due to their excellent sensitivity together with remarkable specificity [13,14]. However, given the low expression of T4 PNKP, the insufficient sensitivity of these approaches with a ratio of one T4 PNKP to one fluorescent signal limited their practical application. Thus, detection methods with higher sensitivity need to be developed. On this basis, some isothermal signal amplification strategies have been proposed, including the hybridization chain reaction [15], catalytic hairpin self-assembly (CHA) [16], and rolling circle amplification [17,18]. Despite their high application efficiency, harsh thermal annealing, expensive protocols and unnecessary by-products hinder their widespread application. Therefore, there is an urgent need to develop more convenient signal amplification strategies for improving the sensitivity of fluorescent sensing platforms.

The clustered regularly interspaced short palindromic repeats (CRISPR) associated protein (Cas) system is regarded as “God’s scissors” and has received a lot of attention due to its programmable gene editing and transcriptional modulation [19,20,21,22,23]. Specifically, its integration of molecular recognition elements and flexible signal transduction modules has made the CRISPR/Cas12a system a potentially programmable platform for building DNA-based signal amplification protocols [23,24]. Recently, a series of CRISPR/Cas12a systems have been demonstrated to possess the capability of specific (cis-cleavage) or non-specific (trans-cleavage) nucleic acid degradation activity on ssDNA [25,26,27,28]. However, rational molecular programming of nucleic acid modules (the guide RNA or DNA activator) in the CRISPRs/12a system would wire up the inherent multifunction of Cas to achieve the autocatalytic generation of active Cas proteins. For example, Peng et al. designed a modular catalytic hairpin assembly circuit to generate double-stranded DNA (dsDNA), which triggered and activated the trans-cleavage activity of CRISPR/Cas12a for further signal amplification [26]. Chen et al. proposed a methodology-based RNA-based catalytic hairpin assembly (CHA) circuit coupled with CRISPR-Cas12a for one-step detection of microRNAs (miRNAs) under isothermal conditions [27]. Wang et al. designed a PNKPP-triggered nicking enzyme-mediated strand displacement amplification reaction to enrich the activator DNA strands for the CRISPR/Cas system [28].

Although some progress has been made regarding fluorescence-based CRISPR/Cas12a biosensors, most research has focused on regulating CRISPR/Cas12a activity by generating a single DNA-activating strand, with less research on its guide RNA [29]. The guide RNAs with small molecular weight often need to be purchased and are at risk of nuclease degradation during storage and utilization, resulting in reduced activation efficiency of CRISPR/Cas12a and inadequate signal amplification. In addition, the harsh thermal annealing, strict stoichiometric ratio of multiple DNA strands, and slow assembly dynamics hamper a more widespread application. T7 RNA polymerase (T7 RNAP) is one of the preferred workhorses for synthesizing RNA molecules in vitro, owing in part to its high transcriptional activity and easily manipulated promoter [30,31]. Because T7 RNAP is highly processable, prone to through-read transcription, and the resulting RNA sequences can be precisely defined by DNA templates, T7 RNAP-RNA is expected to achieve multiple regulation of CRISPR/Cas12a activity by generating multiple guide RNA copies.

Herein, we proposed a self-supplying guide RNA-mediated CRISPR/Cas12a system for the sensitive detection of T4 PNKP, in which multiple copies of guide RNA were generated by in situ transcription. In this assay, T4 PNK was chosen as a model, and a dsDNA probe with T7 promoter region and the transcription region of guide RNA were involved. Under the action of T4 PNK, the 5′-hydroxyl group of the dsDNA probe was converted to a phosphate group, which can be recognized and digested by Lambda Exo, resulting in dsDNA hydrolysis. The transcription template was destroyed, resulting in the failure of the transcription pathway to produce guide RNA. As a result, the CRISPR/Cas12a system could not be activated to effectively cleavage the F-Q-report probe, and the fluorescence signal was turned off. In the absence of T4 PNK, the 5′ -hydroxyl group of the substrate DNA cannot be digested by Lambda Exo. Intact dsDNA acts as a transcription template to produce a large number of guide RNA. Finally, the resulting Cas12a/gRNA complex triggered the reverse cleavage of Cas12a on the F-Q-reporter, producing an “on” fluorescence signal. In our system, the dsDNA simultaneously serves as the T4 PNK substrate, transcription template, and Lambda Exo substrate, avoiding the need for multiple probe designs and saving costs. By integrating the target recognition, Lambda Exo activity, and trans-cleavage activity of CRISPR/Cas12a on the F-Q-reporter, synergetic signal amplification for sensitive T4 PNK detection was enabled. In addition, T4 PNK has been evaluated as a powerful tool in biomedical research and clinical diagnosis in cancer cells, demonstrating good practical application capability.

## 2. Results and Discussion

### 2.1. The Working Principle of the Proposed Method

**Figure 1:**
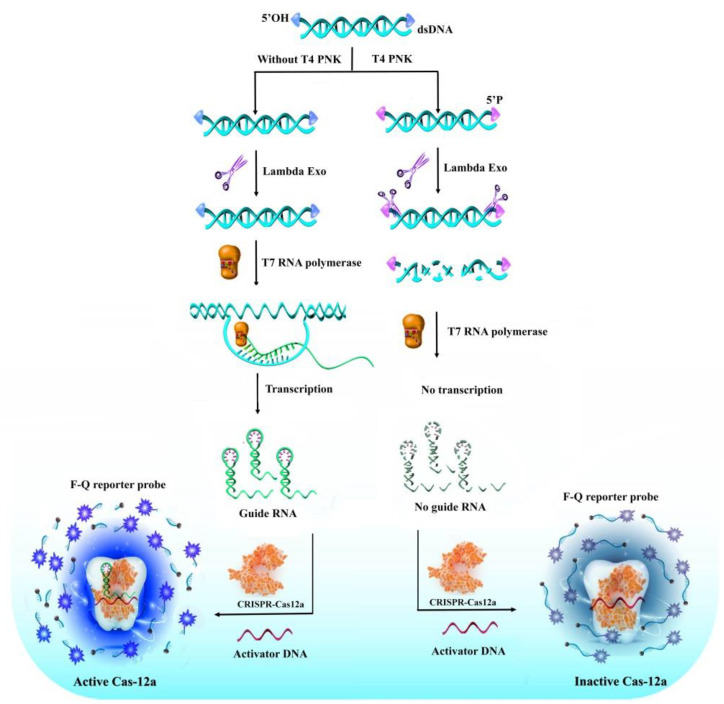
The principle of the self-supplying guide RNA-mediated CRISPR/Cas12a fluorescence system for sensitive detection of T4 PNKP.

The working principle of the self-supplying guide RNA-mediated CRISPR/Cas12a fluorescence system for sensitive detection of T4 PNKP in cancer cells is illustrated in Figure 1. In this assay, notably, a multifunctional double-stranded DNA with hydroxyl group terminal (formed by msg and sg) was designed as a substrate probe. The dsDNA contained two regions: T7 promoter and the transcription region of gRNA. At the same time, it can be used as a substrate template for T4 PNKP recognition. With the addition of T4 PNKP, the 5′-hydroxyl group of the substrate DNA can be transformed to a phosphate group by ATP, which can be recognized and digested by Lambda Exo, resulting in dsDNA hydrolysis. The transcription template was destroyed, which resulted in the failure to generate guide RNA by transcription pathway. Therefore, the CRISPR/Cas12a system could not be activated to effectively cleavage fluorescent-labeled short-stranded DNA probes, and the fluorescence signal was turned off. In the absence of T4 PNKP, the 5′-hydroxyl group of the substrate DNA cannot be digested by Lambda Exo. The intact dsDNA acts as the transcription template to generate a large number of guide RNA. Finally, the formed Cas12a/gRNA complex triggered the reverse cleavage of Cas12a on the F-Q-reporter, resulting in a “turn-on” fluorescence signal.

### 2.2. Feasibility Verification of the Sensing System

**Figure 2:**
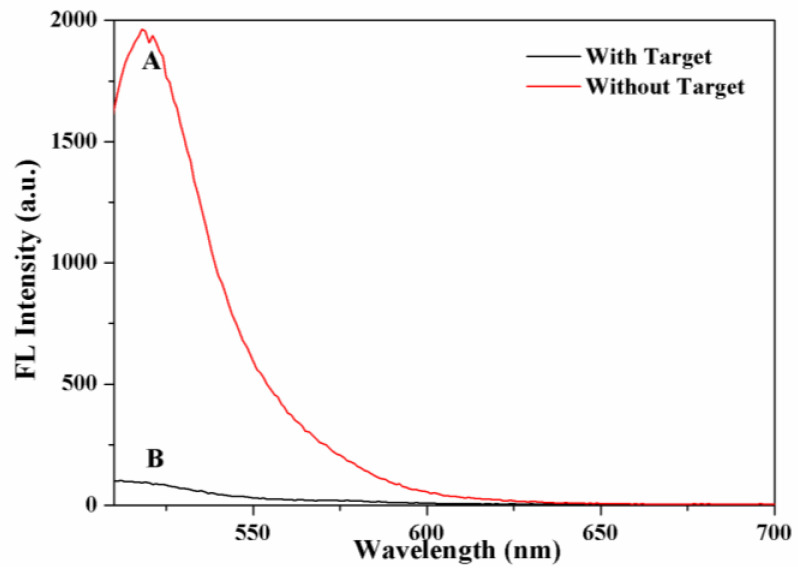
Fluorescence spectra of the proposed T4 PNKP sensor under different conditions: in the presence of T4 PNKP (black curve, B); in the absence of T4 PNKP (red curve, A).

To verify the feasibility of the proposed self-supplied crRNA-regulated CRISPR/Cas12a activity platform to detect T4 PNKP, fluorescence experiments were carried out. As shown in Figure 2, in the absence of T4 PNKP (red curve A), a significant fluorescence signal was obtained clearly. This was because the 5′-OH of dsDNA cannot be recognized and digested by Lambda Exo. The intact dsDNA acts as the transcription template with the action of T7 RNA polymerase, generating multiple guide RNA molecules. Finally, the formed Cas12a/gRNA complex triggered the reverse cleavage of Cas12a on the F-Q-reporter, resulting in the FAM moving away from the BHQ and a significant “turn-on” fluorescence signal. In contrast, when the target T4 PNKP was present, the fluorescence signal was reduced significantly (black curve, B). This indicated that the T4 PNKP causes phosphorylation of dsDNA at the end of 5′, which can be recognized and digested by λ exo specifically. Because dsDNA was destroyed by λ exo, the transcription reaction and the production of guide RNA were blocked, resulting in the suppression of the CRISPR/Cas12a system’s activity. Therefore, the reverse cleavage of the F-Q-reporter was blocked, which prevented the separation of FAM and BHQ, and the fluorescence signal of the system was weakened and entered the shutdown state. Therefore, it is feasible to detect the T4 PNKP by the weakening degree of fluorescence signal.

**Figure 3:**
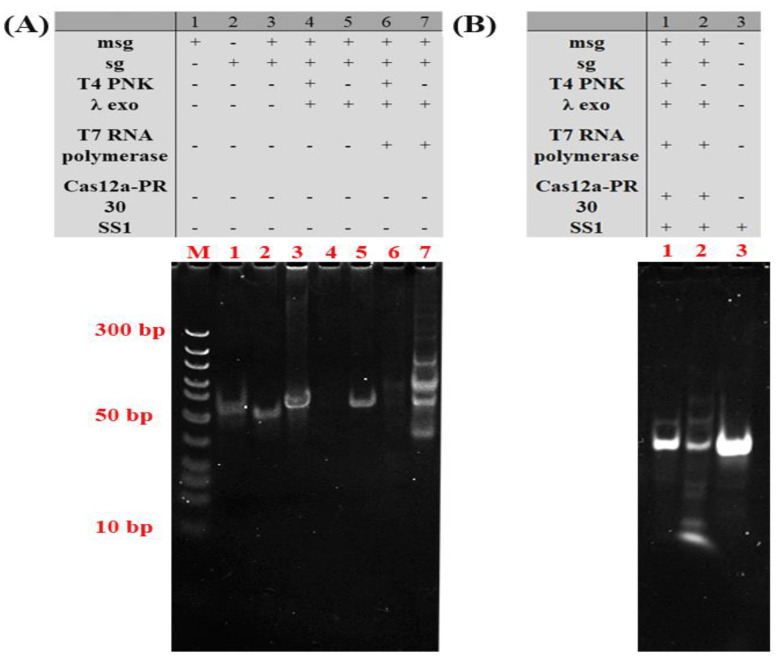
Non-denaturing polyacrylamide gel electrophoresis analysis of the proposed biosensor for T4 PNKP under different conditions. (**A**) Lane M, DNA Marker; Lane (1) msg; (2) sg; (3) msg +sg; (4) msg +sg +T4PNKP+ λ exo; (5) msg +sg + λ exo; (6) msg +sg +T4PNKP+ λ exo+ T7 RNA polymerase; (7) msg +sg + λ exo+ T7 RNA polymerase; (**B**) Lane M, DNA Marker; Lane 1-3: (1) msg +sg +T4PNKP+ λ exo+ T7 RNA polymerase+PR30+SS1; (2) msg +sg + λ exo+ T7 RNA polymerase+PR 30+SS1; (3) SS1.

The performance of the proposed T4 PNKP sensor was also confirmed by the gel electrophoresis, which can reveal what products are being formed as a reaction continues. As shown in Figure 3A, the bands in lane 3 correspond to the newly formed dsDNA (msg and sg) appearing. When T4 PNKP is present (Figure 3A, lane 4), the band corresponding to the newly formed dsDNA disappeared, indicating that the T4 PNKP-based recognition process works. In lane 5 of Figure 3A, the new bright band corresponding to the dsDNA (msg and sg) was present in the case of λ exo. In lane 7 of Figure 3A, a series of new bands with high molecular weight were observed, suggesting that the dsDNA-based transcription process works with the help of T7 RNA polymerase. As shown in Figure 3B, the transcription reaction was blocked in the presence of T4 PNKP. This indicated that the T4 PNKP causes phosphorylation of dsDNA at the end of 5′, which can be digested by λ exo specifically, resulting in the destruction of dsDNA and the blocking of the transcription reaction and the production of guide RNA. After Cas12a-PR-30 and SS1 were added, there was no obvious short chain generation (Figure 3B, lane 1) compared with SS1 (Figure 3B, lane 3) in the control group. The template was degraded, the transcription reaction was blocked and the fluorescence decreased in the presence of T4 PNKP. In the absence of T4 PNKP, SS1was cut (Figure 3B, lane 2). All these results suggest that self-supplied crRNA regulation of the CRISPR/Cas12a activity platform can be used for T4 PNKP analysis.

### 2.3. Optimization of Assay Conditions

**Figure 4:**
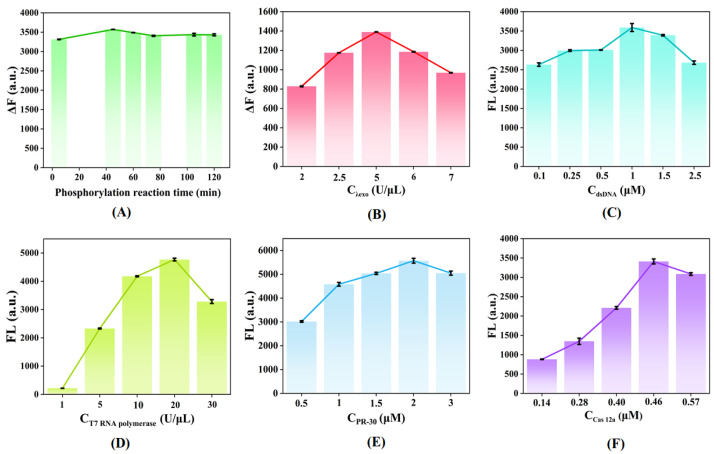
Effect of the (**A**) phosphorylation reaction time, (**B**) λ exo concentration, (**C**) dsDNA concentration, (**D**) T7 RNA polymerase concentration, (**E**) PR-30 concentration, and (**F**) Cas12a concentration on the fluorescence difference. Other reaction conditions: ATP, 10 mM; F-Q Probe, 1 μM. Error bars mean standard deviations (*n* = 3).

To obtain the best assay performance, some parameters that significantly affect transcriptional performance and CRISPR/Cas-12a activity were optimized in detail, including the concentration of dsDNA, PR-30, the reaction time of phosphorylation, the concentration of T7 RNA polymerase, Cas12a, and the λ exo. The change of phosphorylation reaction time directly affects the activity of λ exo and indirectly affects the concentration of the dsDNA in solution. As shown in Figure 4A, when the reaction time reached the maximum value at 45 min, the fluorescence intensity decreased slightly and then plateaued. Thus, 45 min was selected as the optimal reaction time for the phosphorylation process. It was well accepted that the 5′-phosphoryl group of the substrate DNA can be digested by Lambda Exo. As indicated in Figure 4B, the λ exo concentration was optimized. As the λ exo concentration continued to increase, the value of fluorescence gradually increased until the concentration reached 5 U/μL and then decreased, so 5 U/μL was chosen as the optimal λ exo concentration. Moreover, the concentration of dsDNA and the concentration of T7 RNA polymerase were critical for the transcription of guide RNA. As shown in Figure 4C, with the concentration of dsDNA increasing, the intensity of fluorescence clearly changed. When the dsDNA reached 1 μM, the fluorescence intensity reached its highest, and then the fluorescence intensity decreased. Therefore, 1 μM was selected as the optimal concentration. As shown in Figure 4D, when the concentration of T7 RNA polymerase was 20 U/μL, the fluorescence signal intensity gradually reached its peak. Therefore, 20 U/μL was used as the optimal concentration of T7 RNA polymerase. In addition to the guide RNA, rational molecular programming of PR-30 (DNA activator) in CRISPRs/12a system would wire up the inherent multifunction of Cas to achieve the autocatalytic generation of active Cas proteins, which would regulate the activity of the CRISPRs/12a system. Optimizing the PR-30 concentration was essential; with the concentration of PR-30 increasing from 0.5 μM to 3 μM, the fluorescence intensity increased to a maximum at 2 μM (Figure 4D). Furthermore, with the increasing of Cas12a concentration, the fluorescence intensity gradually increased, reached a peak at 0.46 μM, and decreased slightly after reaching 0.46 μM. Thus, 0.46 μM Cas12a concentration was chosen as the optimal condition.

### 2.4. Analytical Performance of the T4 PNKP Detection

**Figure 5:**
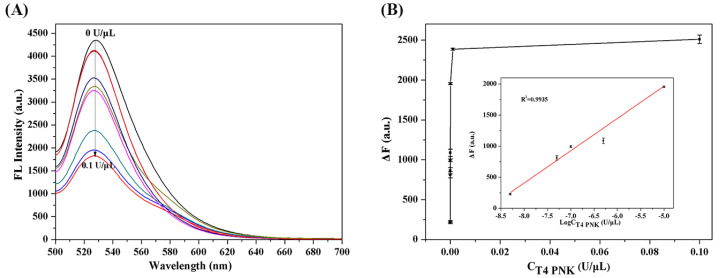
(**A**) Fluorescence spectra measured by different concentrations of T4 PNKP; (**B**) the relationship between the linear range of fluorescence intensity and T4 PNKP concentration. Reaction conditions: dsDNA, 1 μM; ATP, 10 mM; λ exo, 5 U/μL; phosphorylation reaction time, 45 min; T7 RNA polymerase, 20 U/μL; Cas12a, 0.46 μM; PR-30, 2 μM; F-Q, 1 μM. Error bars mean standard deviations (*n* = 3).

Under optimal reaction conditions, the detection of sensitivity and kinetic range of T4 PNKP by the self-supplying guide RNA-mediated CRISPR/Cas12a fluorescence platform proposed were evaluated. As shown in Figure 5A, compared with the fluorescence in the absence of T4 PNKP, with the concentration of T4 PNKP increased from 10^−9^ U/μL to 0.1 U/μL, the fluorescence intensity decreased continuously. As shown in Figure 5B, a good linearity relationship was obtained between fluorescence difference and the logarithm of T4 PNKP concentration with a regression coefficient of R = 0.9935. The limit of detection for this method was calculated to be 1.7 × 10^−6^ U/mL by the triple signal-to-noise method. The LOD was lower than that of most reported T4 PNKP detection strategies (Table 1), which was ascribed to the following reasons. By integrating the target recognition, Lambda Exo activity, and trans-cleavage activity of Cas12a, CRISPR/Cas12a catalyzed cleavage of fluorescent-labeled short-stranded DNA probes, enabling synergetic signal amplification for sensitive T4 PNKP detection.

**Figure 6:**
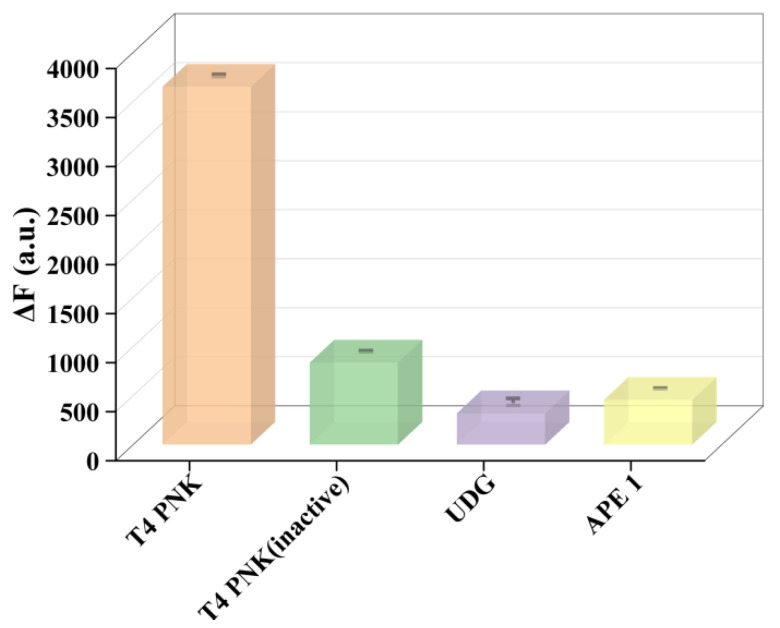
The fluorescent intensity induced by T4 PNK and other alternatives: T4 PNKP, T4 PNKP (inactive), UDG, and APE 1. Error bars mean standard deviations (*n* = 3).

In order to investigate the selectivity of the proposed method for T4 PNKP, Various biological enzymes with similar functions were investigated as interference by detecting their fluorescence reactions, such as inactivated T4 PNKP, the uracil glycosylase (UDG), and the apurinic/apyrimidinic endonuclease (APE 1)e. As shown in Figure 6, a significant fluorescence difference can be clearly detected in the presence of T4 PNKP. In contrast, only a slight change in fluorescence intensity occurs in the presence of the other three enzymes (UDG, APE 1, inactivated T4 PNKP) in comparison to the background. These results suggested that the proposed self-supplying guide RNA-mediated CRISPR/Cas12a fluorescence platform can well distinguish the T4 PNKP from other analogues.

### 2.5. Analysis of Complex Biological Sample

In order to test the possibility of this sensing method in complex biological samples, different concentrations of T4 PNKP in diluted cell lysate samples were investigated. The spiked concentrations of T4 PNKP were 10^−8^ U/μL, 10^−7^ U/μL, and 10^−6^ U/μL, and the recoveries of the spiked sample were in the range of 93.0%–104.0%, with standard deviations of less than 4.0%. These results suggest that the proposed strategy has great potential applicability for detection of T4 PNKP activity in complex biological samples (Table 1).

## 3. Materials and Methods

### 3.1. Reagents and Instrumentation

DNA oligonucleotides (Table 2) were synthesized and purified by Sangon Biotech (Shanghai, China). Cas12a protein (Cas12a) was obtained from Guangzhou Meige Biotechnology Co., Ltd. (Guangzhou, China). T4 polynucleotide kinase (T4 PNKP), Lambda exonuclease (λ exo), T7 RNA polymerase, and rNTP were obtained from New England Biolabs (Beijing, China). The water used in the experiment was ultrapure water (18.2 MΩ·cm), and the other reagents were of analytical grade.

Fluorescence spectra were obtained using a Hitachi F-7100 spectrofluorometer (Hitachi, Japan). Gel electrophoresis images were scanned using the Molecular Imager^®^ GelDoc™ XR+ Imaging System (Bio-Rad, Hercules, CA, USA). The water (18.2 MΩ·cm) used in the whole experiment was pretreated with a Milli-Q ultrapure water treatment system (Millipore, Burlington, MA, USA).

### 3.2. T4 PNKP Activity Assay

The preparation of substrate probes: 2 μM DNA probe (msg) and 2 μM DNA probe (sg) were denatured for 5 min at 90 °C, and then cooled to room temperature gradually to form double-stranded DNA (dsDNA). The phosphorylation reactions were carried out in mixed solutions containing T4 PNKP at different concentrations. Then, 1 μM dsDNA, 3 μL 10 mM ATP, and 10 × T4 PNKP buffer solution were incubated at 37 °C for 45 min. After, 5 U λ exo, 10×λ exo buffer solution, and enzyme-free water were added to digest the phosphorylated dsDNA at 37 °C for 2 h. Finally, the enzymes were inactivated at 75 °C for 10 min. The above reaction solutions were added to 20 U T7 RNA polymerase, 20 mM rNTP, and incubated at 37 °C for 1.5 h. Then, 0.46 μM Cas12a, 2 μM PR-30, and 1 μM F-Q in 1 × Cas12a buffer solution were added for the trans-cleavage reaction at 37 °C for 1 h, and then incubated at 75 °C for 15 min.

### 3.3. Selectivity Assay

The selectivity of this system was studied by using the other three kinds of DNA enzymes (inactivated T4 PNKP, the uracil glycosylase (UDG), and the apurinic/apyrimidinic endonuclease (APE 1)) as interfering enzymes. The target T4 PNKP was replaced by the three kinds of DNA enzymes, and the following procedures were performed in the same way as in the T4 PNKP activity assay.

### 3.4. Polyacrylamide Gel Electrophoresis Analysis

A 15% non-denatured polyacrylamide gel electrophoresis (PAGE) experiment was performed to prove the feasibility of the developed biosensor. The obtained production was analyzed in 1×TAE buffer at 120 V constant voltage for 1.5 h. After electrophoresis, the gel was stained in ethidium bromide (EB) for 5 min and was photographed by a UV imager system.

### 3.5. T4 PNKP Activity Detection in Diluted Cell Extracts

Hela cells (1.0 × 10^5^) were placed in 1.5 mL EP tubes, rinsed twice with cold PBS (0.1 M, pH 7.40), and then resuspended in 200 μL CHAPS lysis buffer. The CHAPS buffer contained 10 mM Tris-HCl, pH 7.50, 1 mM MgCl_2_, 1 mM EGTA, 0.1 mM PMSF, 0.5% CHAPS, and 10% glycerol. The mixture was incubated for 30 min on ice and centrifuged at 16,000 rpm at 4 °C for 20 min. The supernatant was collected as cell extract for analysis. Dilute cell extracts were added to the assay solution. The procedure is the same as described above.

## 4. Conclusions

In conclusion, integrated target recognition, Lambda Exo activity, and trans-cleavage activity of Cas12a strategy were developed for sensitive and specific detection of T4 PNKP in cancer cells. This proposed strategy achieves a sharper sensitivity of T4 PNKP than that of most previously reported methods, and well distinguishes T4 PNKP from the other analogues. In our system, the dsDNA simultaneously serves as the T4 PNKP substrate, transcription template, and Lambda Exo substrate, avoiding the need for multiple probe designs and saving costs. By integrating the target recognition, Lambda Exo activity, and trans-cleavage activity of Cas12a, CRISPR/Cas12a catalyzed the cleavage of fluorescent-labeled short-stranded DNA probes, enabling synergetic signal amplification for sensitive T4 PNKP detection. Importantly, the detection of T4 PNKP in cancer cells was performed, suggesting that the proposed approach was reliable and had a great capacity for medical research and early diagnosis. Although it was demonstrated well in the lysate of cancer cells, the limitation of this study was that it did not focus on the activity of PNKP in living cells. It is expected that the real-time visual detection of PNKP activity in living cells can be achieved in the future.

## Figures and Tables

**Table 1:** Comparison of T4 PNKP detection methods.

Method	Linear Range	LOD	Ref.
Photoelectrochemical	0.1~20 mU/mL	0.069 mU/mL	[12]
Fluorescence	50~1500 mU/mL	5 mU/mL	[14]
Electrochemical	10~5000 mU/mL	8.9 mU/mL	[15]
Electrochemical	0.00001~20 U/mL	3.0 × 10^−6^ U/mL	[17]
Fluorescence	1~100 mU/mL	0. 34 mU/mL	[19]
Fluorescence	0.01~25 mU/mL	0.0033 mU/mL	[28]
Fluorescence	0.001~0.5 mU/μL	0.0002 mU/μL	[32]
Fluorescence	0.00001~0.01 U/mL	8.1×10^−6^ U/mL	[33]
Fluorescence	0.00008 to 0.1 U/m	1.8 × 10^−5^ U/mL	[34]
Fluorescence	0.001 to 0.05 U/mL	6.63 × 10^−4^ U/mL	[35]
Photoelectrochemical	10^−4^~1 U/mL	6 × 10^−5^ U/mL	[36]
Fluorescence	0.005~1 mU/mL	0.0017 mU/mL	This work

**Table 2:** Sequences of oligonucleotides used in this study.

Name	Sequence (5′-3′)
msg	CGC CTT ATT AGA TGA CTT CTC ATC TAC ACT TAG TAG AAA TTA CCC TAT AGT GAG TCG TAT TA
sg	TAA TAC GAC TCA CTA TA GGG TAA TTT CTA CTA AGT GTA GAT GAG AAG TCA TCT AAT AAG GCG
PR-30	TGA GGC GCC TTA TTA GAT GAC TTC TCT AAA
F-Q	6-FAM-TTA TT-BHQ1
SS1	TAMRA-TTT TCT CAT ACC ACT GCT CAT CCA TGC CTA GAC TGG CGA TAA GTA GCC AGC

## Data Availability

The data presented in this study are available upon request from the corresponding author.
